# Notes and News

**Published:** 1895-03-16

**Authors:** 


### Notes and News.

Collected and Communicated.

The Council of the Hospital Saturday Fund have decided to hold their annual ladies' street collection on Saturday, July 13th.

Mr. E. Wilson Taylor has resigned the secretaryship of the London Temperance Hospital, which he has held since 1890, having received an important commercial appointment.

H.R.H. the Duchess of York, attended by Lady Eva Greville, visited the Chelsea Hospital for Women on Monday, and expressed herself highly pleased and interested with everything she had seen. Her Royal Highness distributed flowers to all the patients in the hospital, and signed her name in the visitors' book on leaving. Her Royal Highness was escorted round the wards by Miss Heather-Bigg, the matron, and some of the medical staff. The wards were tastefully arranged for the occasion.

In Dr. Alphonse Guérin, who has recently passed away, France has lost one of her most prominent surgeons. He struck out a line of his own in the treatment of wounds long before the antiseptic method had taken root in surgery. Guérin's contributions to medical literature were numerous and important. He held many honourable and responsible posts, amongst them being the presidency of the Academies of Medicine and Surgery. He was a man of fine personal character.

The proceedings of the German Medical Congress will be of exceptional general interest, as the main subject under discussion is to be the results of the anti-toxin treatment for diphtheria. Since the Congress at Buda Pest, in the autumn, when this treatment received so great an impetus, a large amount of experience has become available for comparative observations, and not only should a better understanding as to strength of dose be arrived at, but the precautions necessary to secure a reliable medium should be more definitely ascertained. Professors Heubner and Baginsky, of Berlin, will bring forward the subject at the Congress, which is to be held at Munich on April 2nd.

We are glad to learn that Sir Henry Acland's wishes as to the form of memorial to be raised in his honour is to be respected. To do otherwise would be to disregard the primary intention of his friends and admirers, which is to afford gratification to the eminent physician.

The annual report of the Life Saving Society shows good work accomplished in various parts of Great Britain and the Colonies. The object of the society is to promote technical education in life saving, the resuscitation of the apparently drowned, &c. No less than 334 students have passed the examination during the year.

The yearly reports of hospitals and charitable institutions in the colonies shows us that the low price of products, and the general agricultural depression in New Zealand, are grave reasons for fearing that a considerable increase in charitable expenditure will be required. The way in which this emergency was met in Wellington demonstrates that the important question of outdoor relief can never be satisfactorily dealt with till it is met directly by the people themselves. Three private gentlemen (of Wellington) raised in a few days a sum of £600, which, with a further sum contributed by the City Council, was, in an entirely unconditional manner, handed over to the Benevolent Society. The money thus collected was being distributed in a careful and intelligent manner amongst those most in need. All got enough to live upon, though no idle loafing was tolerated; no man being assisted through these funds who was able to work and refused to do so. This is a fairly good example of the excellent results in out-door relief being expended by local bodies.

We have received from Mr. Leet, F.R.C.S., of Liverpool, some interesting papers relating to his endeavour to obtain the establishment of a medical department of the Board of Trade, presided over by a chief officer who would be charged with the duty of seeing that proper provision was made for the sanitation of merchant shipping, and that the existing regulations concerning vision testing in the merchant navy, with regard both to form vision and to colour vision, were strictly enforced, and, when necessary, amended from time to time. There can be no doubt that great neglect, and, still more great ignorance, prevail with reference to both of these important requirements; and the appointment of a responsible medical officer would, unquestionably, afford one way of mitigating evils which now exist. But "Government inspection," however dear to the official mind, is not universally looked upon as an unfailing panacea for all the evils to which flesh is heir. If Mr. Leet could dissipate the ignorance which stands in the way of effecting the improvements which he has at heart, it seems not improbable that a more enlightened action would be found to appeal to the self-interest of shipowners and shippers, not to speak of passengers; and that, even without an inspector, the enforcement of proper conditions of living on shipboard, and of proper visual tests, would bring advantages in the struggle for existence which is carried on in all businesses alike.

